# Long-Term Results of a Minimally Invasive Surgical Pulmonary Vein Isolation and Ganglionic Plexi Ablation for Atrial Fibrillation

**DOI:** 10.1371/journal.pone.0079755

**Published:** 2013-11-11

**Authors:** Shuai Zheng, Yan Li, Jie Han, Haibo Zhang, Wen Zeng, Chunlei Xu, Yixin Jia, Jiangang Wang, Kequan Guo, Yuqing Jiao, Xu Meng

**Affiliations:** Department of Cardiac Surgery, Atrial Fibrillation Center, Beijing Anzhen Hospital, Capital Medical University, Beijing, China; University Heart Center, Germany

## Abstract

**Background:**

Ganglionated plexi (GP) ablation has been become an adjunct to pulmonary vein isolation (PVI). This study describes the long-term results of minimally invasive surgical PVI, ablation of GPs, and exclusion of the left atrial appendage for atrial fibrillation (AF).

**Methods:**

Long-term follow-up of 55 months was performed in 139 consecutive patients (age 58.3±20.8 years) with symptomatic, drug-refractory lone AF who underwent minimally invasive surgical PVI, GPs ablation, and exclusion of the left atrial appendage. Success was defined as freedom from AF, atrial flutter, or atrial tachycardia off antiarrhythmic drugs.

**Results:**

AF was paroxysmal in 77.7%, persistent in 12.2% and long-standing persistent in 10.1%. Single-procedure success rate was 71.7%, 59.4% and 46.6% at 12, 24 and 60 months respectively. Single-procedure success rate was 72.9%, 62.6% and 51.8% for paroxysmal AF, 64.7%, 35.3%, and 28.2% for persistent AF, 71.4%, 64.3% and 28.6% for long-standing persistent AF at 12, 24 and 60 months respectively. Duration of AF>24 months (hazard ratio [HR]: 3.09, 95% confidence interval [CI]: 1.51 to 6.32; p = 0.002), left atrial diameter≥40 mm (HR: 4.03, 95% CI: 1.88 to 8.65; p<0.001), early recurrence of AF (HR: 4.66, 95% CI: 2.25 to 9.63; p<0.001) independently predicted long-term recurrence of AF. There was no procedure-related death. One patient converted to median sternotomy because of uncontrolled bleeding. Two patients underwent perioperative cerebrovascular events.

**Conclusions:**

At nearly 5-year of clinical follow-up, single-procedure success rate of minimally invasive surgical PVI with GP ablation was 51.8% for paroxysmal AF, 28.2% for persistent AF, 28.6% for long-standing persistent AF after initial procedure. Patients with AF duration≤24 months, left atrial diameter<40 mm and no early recurrence of AF, had favorable outcomes.

## Introduction

Cox-Maze III procedure has been regarded as the gold standard for the treatment of AF, but it did not achieve widespread use primarily due to its complexity and invasive essence [1]. With the development of surgical technology, minimally invasive surgery has emerged as an alternative to the Cox Maze III procedure for the treatment of AF [2]. Haissaguerre et al [3] revealed that ectopic beats from pulmonary veins (PV) may trigger AF, which lead to the development of catheter-based and surgical AF ablation with the goal of electrical isolation of bilateral PV [1].

Cardiac autonomic nervous and ganglionated plexi (GP) play an important role in the trigger and maintenance of AF [4], [5]. Now GP ablation has been become an adjunct to pulmonary veins isolation (PVI) [6], [7]. Though attempting to ablate the left atrial (LA) GP through minimally invasive surgical approach has produced promising short-term results [8], [9], [10], the long-term results of it has not been reported.

We have previously described the minimally invasive technique for the treatment of AF [11], [12]. In the present study, we investigated the long-term results after minimally invasive surgical PVI, ablation of GPs, and exclusion of the left atrial appendage (LAA) in patients with AF.

## Materials and Methods

### Patients

Between December 2006 and September 2009, 139 consecutive patients with lone AF underwent minimally invasive surgical PVI combined with GP ablation in our hospital. Indications were symptomatic drug-refractory lone AF, including paroxysmal, persistent and long-standing persistent AF. The exclusion criteria were LA size of greater than 70 mm, left ventricular ejection fraction of less than 30%, previous cardiac surgery, and severe pleural adhesions. Patients who underwent previous catheter ablation were eligible for this study. All definitions and reporting standards were in accordance with the Heart Rhythm Society/European Heart Rhythm Association/European Cardiac Arrhythmia Society Expert Consensus Statement [1]. The study was approved by the institutional review board of Anzhen Hospital, Capital Medical University, Beijing. All participants in this study gave a written informed consent.

### Preoperative Management

Laboratory tests, 12-lead ECG, chest radiography, transthoracic and transesophageal echocardiogram were performed on admission. Computer tomography scanning or coronary angiographic analysis was performed on indication. Oral anticoagulation was discontinued 5 days before surgery. Antiarrhythmic drugs (AAD) were continued after admission.

### Surgical Technique

The procedure is performed after achievement of general anesthesia administrated with a double-lumen endotracheal tube. The procedures has been previously reported by Dr Randall Wolf[2], which including bilateral thoracotomy, epicardial GP ablation and radiofrequency PVI, LAA excision, ligament of Marshall ablation and electrophysiologic testing. Procedure was performed on the right side first.

GPs were detected by high-frequency stimulation (10 V, 800 Hz with a pulse width of 9.9ms) as described previously [13]. Stimulation resulting in an increase in the RR interval of greater than 50% from baseline was deemed to represent a location of active GP. Active GPs were subsequently ablated with bipolar radiofrequency energy. Elimination of a vagal response to restimulation confirmed GP ablation.

Blunt dissection around the PV was conducted by the AtriCure Lumitip Dissetor (AtriCure, Inc., West Chester, Ohio, USA), a bipolar radiofrequency clamp and regenerator system (AtriCure, Inc., West Chester, Ohio, USA) were used to achieve linear and transmural ablation lesion. The end point for PV ablation was complete entrance and exit block into and from the PVs as we previously described [11], [12]. The epicardial pacing leads are routinely placed. After confirmation of right PVI, the procedure was repeated on the left side. The ligament of Marshall was dissected by electrocautery under direct vision. The LAA was removed with EZ-45G Endostapler (Johnson and Johnson Medical, Inc, Arlington, Tex) after the left PVI. On completion of the procedure, a chest tube were placed; one in each side of the chest. Multiple ribs were blocked with 1% lidocaine.

### Postoperative Medical Management

Antiarrhythmic drugs were usually continued after surgery. Amiodarone was administered to patients at approximately 100 to 200 mg/d for 3 months and then tapered off in the presence of a stable sinus rhythm (SR). Meanwhile, a β-blocker was served as rate-control medication according to postoperative heart rate. Postoperative anticoagulation was in accordance with the instructions found in the American College of Cardiology/American Heart Association/European Society of Cardiology guidelines [14]. Electrical cardioversion (ECV) was recommended if a patient had symptomatic AF lasting for more than 24 hours.

### Follow-up

Follow-up was obtained from office visits at an outpatient building, and mailed medical records received from local hospitals. The first 3 months after operation was designed as blanking period. Over the first year, 12-lead ECG, 24-hour Holter monitoring, and transthoracic echocardiogram were systematically scheduled at discharge and 1, 3, 6 and 12 months postoperatively. After one year, patients were seen every 6 months at the outpatient clinic. Between visits, patients were encouraged to provide documentation of 12-lead ECG for any suspicious AF. The definition of success was defined as freedom from AF, atrial flutter or atrial tachycardia off AAD. Recurrences that occurred in the 3-month blanking period were classified as early recurrence of AF (ERAF).

### Statistical Analysis

Statistical analyses were performed with SPSS 13.0 software (SPSS, Inc, Chicago, USA). Categorical variables are expressed as the number of cases and percentage, and continuous data are presented as mean ± standard deviations or median and range. Numeric data were compared using the Student's t test or nonparametric test. Event-free survival was estimated according to the Kaplan-Meier method. Univariate and multivariate predictors of recurrent AF were performed using Cox proportional hazards regression models. The prognostic values of LA diameter and AF duration for AF recurrence were assessed by receiver operating characteristics (ROC) curves. Areas under curves (AUC) of greater than 0.7 are generally thought to be useful. The cut-offs were directly estimated using Youden index (sensitivity+specificity – 1) [15]. Then patients were divided into two groups according to cut-offs respectively, difference between groups were compared by the log-rank test.

## Results

### Patient characteristics

The mean age of the patients was 58.3±20.8 (range 29-80) years. Five patients underwent previous percutaneous catheter ablation for AF, two patients for paroxysmal supraventricular tachycardia. Eighty five (61.2%) patients had valvular heart disease. Among these 85 patients, 63 patients suffered from mild mitral regurgitation, 11 patients had mild aortic regurgitation, 9 patients suffered from mild tricuspid regurgitation and 2 patients with moderate tricuspid regurgitation. Patient characteristics are detailed in **Table 1**.

**Table 1:** Patient characteristics.

Characteristics	n = 139
Age, y	58.3±20.8
Male sex, n (%)	91(65.5%)
Body mass index, kg/m^2^	25.4±6.3
Current smoker, n (%)	28(20.1%)
Hyperlipemia, n (%)	18 (12.9%)
Hypertension, n (%)	56 (40.3%)
Diabetes, n (%)	11 (7.9%)
Peripheral vascular diseases, n (%)	1 (0.7%)
Thyroid disease, n (%)	8 (5.8%)
Cardiomyopathy	7 (5%)
Coronary artery disease	20 (14.4%)
Valvular heart disease	85 (61.2%)
Previous PCI	7 (5%)
Previous PBMV	4 (2.9%)
Congestive heart failure, n (%)	0 (0%)
Previous stroke, n (%)	9 (6.5%)
Pacemaker	3 (2.2%)
AF type, n (%)	
Paroxysmal	108 (77.7%)
Persistent	17 (12.2%)
Long-standing persistent	14 (10.1%)
Total duration of AF, months	48 (1-360)
Previous CA, n (%)	7 (5%)
NYHA function class, n (%)	
I	33 (23.7%)
II	100 (71.9%)
III	6 (4.3%)
Preoperative medication, n (%)	
Aspirin	9 (6.5%)
Warfarin	4 (2.9%)
β-blocker	22 (15.8%)
ACEI	41 (29.5%)
ARB	9 (6.5%)
Digitalis	5 (3.6%)
Amiodarone	102 (73.4%)
Propafenone	1 (0.7%)
LVEF	63.5±15.3
LA diameter, cm	40.1±12.5

ACEI = angiotensin converting enzyme inhibitor; AF = atrial fibrillation; ARB = angiotensin receptor blocker; CA = catheter ablation; CCB = calcium channel blocker; LA = left atrium; LVEF = left ventricular ejection fraction; NYHA = New York Heart Association; PBMV = percutaneous balloon mitral valvuloplasty; PCI = percutanous coronary intervention.

### Perioperative results

PVs were successfully isolated and bidirectional block was achieved in all patients. Vagal response could not be evoked by high-frequency stimulation after surgery in all of the patients. The LAA also could successfully be removed in all patients. The blood loss during surgery was about 50 ml (range 20-500 ml). Three patient's blood loss achieved 500 ml because of pulmonary hemorrhage during separating the adhesion of pericardium, and one patient's blood loss achieved 2000 ml because of LAA rupture and who underwent a conversion to median sternotomy. Two patients underwent reexploration for bleeding. There were no procedure-related deaths. One patient aged 70 years had a stroke on the fifth day and died on the 27th day after surgery. This patient had history of hypertension and cerebrovascular events. A patient aged 62 years underwent pulmonary thromboembolism, suffered respiratory insufficiency on the third day after surgery, recovered and discharged at 32th day postoperatively. Three patients had LAA removed before ablation because preoperative transesophageal echo indicated abnormal density in the LAA. All of the 3 patients were confirmed to have thrombus after LAA excision, and no patients experienced perioperative stroke. In total, six patients prolong hospital length of stay because of early complication. In hospital results are presented in **Table 2**.

**Table 2:** Perioperative results.

Perioperative details	n = 139
Procedure time, h	3 (2-6)
LAA removed, n (%)	139 (100%)
Blood loss, ml	50 (20-500)
Conversion to sternotomy, n (%)	1 (0.7%)
Mortality, n (%)	1 (0.7%)
Duration of pleural drainage, d	1.5 (1-4)
Volume of pleural drainage, ml	340 (40-1500)
Ventilation time, h	14 (1-51)
ICU time, h	18 (0-41)
Hospital length of stay, d	7 (4-32)
AF/AFL during hospitalization, n (%)	62 (44.6%)
ECV during hospitalization, n (%)	28 (20.1%)
Complications, n (%)	6 (4.3%)
PTE	1 (0.7%)
Stroke	2 (1.4%)
Pleural effusion	1 (0.7%)
Reexploration for bleeding	2 (1.4%)
Sinus rhythm on discharge, n (%)	127(91.4%)

AF = atrial fibrillation; AFL = atrial flutter; ECV = electric cardioversion; GP = ganglionated plexi; ICU = intensive care unit; LAA = left atrial appendage; PTE = pulmonary thromboembolism.

### Follow-up results

**Figure 1:**
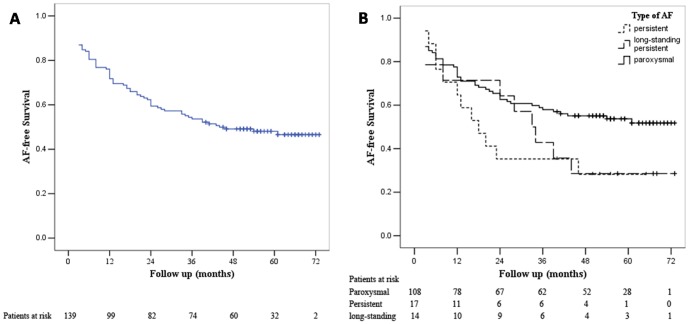
Long-term freedom from AF after single-procedure. (A) overall patients (B) patients specified by type of AF. Plus sign (+) indicates censored.

Patients were followed for a median of 55 months (range 3 to 73 months) from the initial procedure. Sixty four (46.4%) patients had ERAF. Sixty two patients (45%) had an in-hospital relapse of AF, 28 patients received ECV during hospitalization. Thirty two patients received an ECV post-discharge; among these patients 14 patients underwent more than twice ECV post-discharge. Holter-monitoring was available in 91% of the patient who was free from AF. As shown in **Figure 1A**, single-procedure success rates were 71.7%, 59.4% and 46.6% at 12, 24 and 60 months respectively. AF recurred in 39 of 138 (28.3%) patients within the first year after surgery. Thirty three (23.9%) patients had recurrences after 12 months, and 8 of 33 patients (5.8%) recurred after 3 years. Single-procedure success rate were 72.9%, 62.6% and 51.8% for paroxysmal AF, 64.7%, 35.3%, and 28.2% for persistent AF, 71.4%, 64.3% and 28.6% for long-standing persistent AF at 12, 24 and 60 months respectively (**Figure 1B**). There was no statistically significant difference in the risk of failure among the paroxysmal AF, persistent AF and long-standing persistent AF (All p>0.05, **Figure 1B**).

Twelve patients with recurrent arrhythmia (9 AF, 3 AFL) underwent an electrophysiology study and catheter ablation. Three of the twelve patients underwent twice catheter ablation because of recurrent AF (**Table 3**). During a median follow-up of 42 months (range 9 to 59 months), 10 of 12 (83.3%) patients remained in SR after the last procedure. Other two patients with recurrent arrhythmia was both persistent AF before initial surgery. Freedom from AF after the last catheter ablation procedure was 100%, 100%, and 65.6% at 12, 24, and 59 months, respectively. For paroxysmal AF, multiple-procedure success rate was 100% during a median follow-up of 43 months.

**Table 3:** Characteristics of patients underwent catheter ablation after surgery.

Patient	Type of AF	TwiceCA	Follow up (months)	Present rhythm
1	Paroxysmal AF	No	59	SR
2	Paroxysmal AF	No	56	SR
3	Paroxysmal AF	No	48	SR
4	Paroxysmal AF	No	44	SR
5	Paroxysmal AF	No	42	SR
6	Paroxysmal AF	Yes	37	SR
7	Paroxysmal AF	No	23	SR
8	Paroxysmal AF	No	9	SR
9	Persistent AF	No	48	AFL
10	Persistent AF	No	39	AF
11	Persistent AF	Yes	33	SR
12	Long-standing persistent AF	Yes	42	SR

AF = atrial fibrillation; AFL = atrial flutter; CA = catheter ablation; SR = sinus rhythm

### Predictors of arrhythmia recurrence

Gender, age, BMI, smoke, diabetes, hyperlipidaemia, hypertension, cerebrovascular disease history, cardiomyopathy, New York Heart Association Functional class, thyroid disease, previous catheter ablation for AF, coronary artery disease, left ventricular ejection fraction, valvular heart disease and type of AF could not predict recurrence of AF (all p>0.05) in Cox regression analysis.

**Figure 2:**
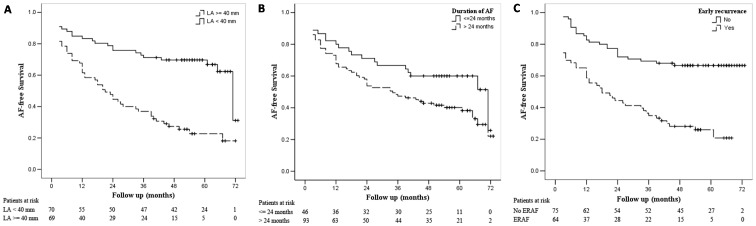
Single-procedure success for patients with LA diameter<40 mm versus≥40 mm (A), with AF duration≤24 months versus>24 months (B), with ERAF versus without ERAF (C). Plus sign (+) indicates censored.

**Figure 3:**
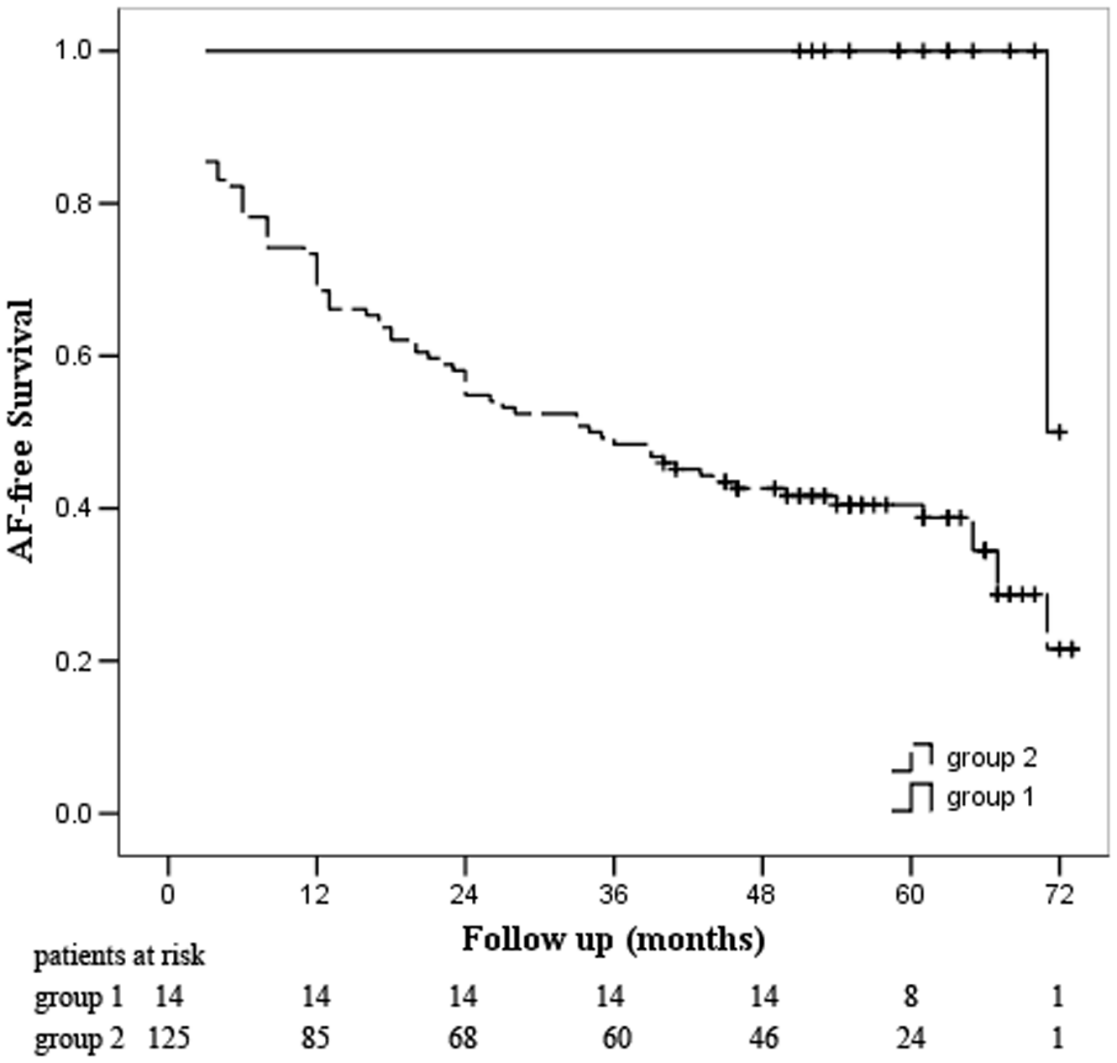
Kaplan-Meier analysis of long-term freedom from AF after the initial procedure. group 1: patients with LA diameter<40 mm and AF duration≤24 months and without ERAF; group 2 patients with LA diameter≥40 mm, AF duration>24 months and with ERAF. Plus sign (+) indicates censored.

Univariate predictors of long-term recurrent AF are duration of AF, LA diameter, ERAF and presence of AF at discharge (**Table 4**). Multivariate Cox regression analysis revealed that duration of AF, LA diameter, ERAF independently predicted recurrences of AF (**Table 4**). The ROC analyses further illustrated that the LA diameter and AF duration were the indicator of recurrent AF, with an AUC of 0.745 (p<0.001) and 0.76 (p<0.001) respectively. The cut-off of LA diameter and AF duration were 39.5 mm and 25 months respectively. **Figure 2** show that patients with AF duration>24 months, LA diameter≥40 mm or ERAF has higher rate of recurrence of AF (All p<0.01). Fourteen patients had an AF duration≤24 months and LA diameter<40 mm and no ERAF, who had excellent rhythm outcome with single-procedure success rate of 100% at 60 months after surgery (p <0.001; **Figure 3**).

**Table 4:** Predictors of long-term recurrence of AF.

Predictors of recurrence	HR, 95%CI	p
Univariate analyses		
Duration of AF	1.29 [1.04, 2.08]	0.045
LA diameter	1.09 [1.05, 1.13]	<0.001
Intraprocedural AF termination	0.51 [0.27, 0.97]	0.04
presence of AF at discharge	2.53 [1.21, 5.29]	0.014
ERAF	3.21 [1.97, 5.24]	<0.001
Multivariate analyses		
Duration of AF	1.60 [1.12, 3.04]	0.03
Duration of AF>24 m	3.09 [1.51, 6.32]	0.002
LA diameter	1.10 [1.04, 1.16]	0.001
LA diameter ≥40 mm	4.03 [1.88, 8.65]	<0.001
ERAF	4.66 [2.25, 9.63]	<0.001

AF = atrial fibrillation; CI = confidence interval; ERAF = early recurrence of atrial fibrillation; HR = hazard ratio; LA = left atrial

### Complications

Two patients died of cancer 3 years after surgery. One patient occurred sudden cardiac death at 12 months after surgery, another patients died of trauma at 58 months after surgery. During follow-up period 5 patients suffered from stroke 2, 4, 21, 26 and 35 months after surgery respectively. Among patients with stroke, one patient with hypertension had recurrence of AF but did not receive any anticoagulation or antiplatelet drug. Two patients with CHADS_2_ score greater than one only received antiplatelet therapy with asprin. Only two patients received regular anticoagulation therapy in accordance with guidelines for AF. Four patients required new postoperative permanent pacemaker implantation for chronotropic incompetentce and bradycardia related to medicatin with beta-blocking agents at 13, 25, 39 and 42 months after surgery, respectively. And one patient with recurrent AF combined with mitral regurgitation and tricuspid regurgitation underwent repeated AF radiofrequency during combined heart valve surgery 40 months after initial procedure.

## Discussion

The present study of 139 patients undergoing a minimally invasive surgical PVI and GP ablation for AF found that during a median follow-up period of 55 months after the initial procedure, (1) AF-free survival was 51.8% for paroxysmal AF, 28.2% for persistent AF, 28.6% for long-standing persistent AF at 60 months; (2) duration of AF>24 months, LA diameter≥40 mm and ERAF independently predicted long-term recurrences of AF.

### Rhythm outcomes

Studies [8], [10], [16] showed that estimated incidence rate of ERAF after PVI is about 30% to 60% during the blanking period. And most recurrence occurs in the first year following initial procedure. In our study, 46.4% patients had an ERAF and 54.2% of (39/72) AF recurred within the first year. In our study, we report the long-term outcome of minimally invasive surgery for AF combined PVI and GP ablation. Single-procedure success rate was 71.7%, 59.4% and 46.6% at 12, 24 and 60 months respectively. We found a similar 1-year success rates to prior surgical reports of 65% to 77% [10], [17], [18], [19].

The first randomized clinical trial comparing catheter ablation with surgical ablation demonstrated that surgical ablation had higher success rate after 12 months than catheter ablation [19]. In theory, surgical ablation may be more efficacious than catheter ablation because of better quality of ablation lesion, effective epicardial GP ablation and LAA excision. However, recently studies [20], [21] reported the long-term results of catheter ablation for AF. Single-procedure success rate was 45.3% for paroxysmal AF [20], 20% for long-standing persistent AF [21] during 5-year follow-up. Additionally, recently meta-analysis [22] showed that single-procedure success at long-term follow-up was 54.1% in paroxysmal AF. According to our results, it seemed that surgical ablation did not have significant advantages over catheter ablation. Higher rate of preoperative comorbidity such as hypertension and valvular heart disease in our patients may account for this. Furthermore in the meta-analysis [22], 12 of 19 studies was retrospective analysis and substantial heterogeneity was significant, which might produce precise but spurious results. However, it seemed that surgical ablation had higher success rate after the last procedure, especially in paroxysmal AF. Weerasooriya et al [24] found arrhythmia-free survival following the last catheter ablation was 87%, 81%, and 63% at 1, 2, and 5 years, respectively. Ouyang et al [20] reported that for paroxysmal AF, during a median follow up of 4.6 years stable SR was achieved in 79.5% patients after the last procedure. In our study, SR was achieved in 100% for paroxysmal AF after the last ablation during a median of 43 months follow up. Hybrid surgical and catheter ablation, a novel technique for the treatment of AF, had achieved better results [23]. Nevertheless, lots of problems still exist in simultaneous hybrid procedure [24], and stage hybrid surgical and catheter ablation seems more feasible. Surgery and catheter which first, maybe our data can provide some evidence. In future, larger multicenter trials are needed to comparing efficacy and safety of surgical and catheter ablation.

### GP ablation

GPs may play an important role in the trigger and maintenance of AF [4], [5]. Pappone et al [6] first reported that compared with patients without a vagal response, patients with vagal response had a higher success rate during catheter ablation. Recent meta-analysis demonstrated that whether for surgical [25] or catheter ablation [25], [26], additional GP ablation to PVI is superior to PVI without GP ablation in short-term results. However, long-term superiority of PVI with GP ablation is not clear up to now. Unfortunately, our study only describe the long-term results of minimally invasive surgical PVI and GP ablation, which has nothing to do with comparing long-term results between PVI with GP ablation and PVI without GP ablation. As we know, the GPs are mainly localized at sites around the circumference of the LA-PV junction. When performing PVI with bipolar radiofrequency clamp, part of the atrial fat pads and GPs are clamped between the jaws. Therefore, it is difficult to compare GP ablation in combination with PVI with GP ablation with absolute PVI alone. Furthermore, whether the effect of GP ablation is persistent is controversial, because atrial reinnervation after ablation may exist [27]. Therefore, short-term superiority of additional GP ablation does not necessarily indicate long-term superiority, and we also cannot answer whether additional GP ablation is beneficial or not for long-term results.

### Predictors of recurrence of AF

The present study demonstrates that duration of AF>24 months, ERAF, LA diameter≥40 mm were independent predictors of long-term recurrences of AF. Though persistent AF and long-standing persistent AF which exacerbate atrial remodeling are difficult to treat, we found type of AF was not a predictor of AF recurrence which was in keeping with previous publications [10], [17], [18]. Previous investigators have identified that duration of preoperative AF was a significant predictor of late recurrence of AF [28]. We also found that duration of AF above 24 months independently predicted recurrence of AF. Atrial dilatation reflects atrial remodeling which is a cause of AF, and larger left atrium has higher rate of recurrence of AF [29]. In the present study we get similar results. Traditionally ERAF has been considered to be a result of the resumption of early electrical activity in ablation lines [30]. ERAF after ablation are associated with a lower long-term success rate than in patients without ERAF [16]. And our study revealed that patients with ERAF has higher rate of recurrence of AF.

### Complications

A small number of patients in our study underwent postoperative adverse events. One patient (0.7%) underwent sternotomy because of uncontrolled bleeding, which is in agreement with the 0-10% estimated risk for patients undergoing minimally invasive surgical PVI and GP ablation [8], [10], [17]. There were no procedure-related deaths in our patient population. And previous studies also indicated that the prevalence of in-hospital mortality of minimally invasive surgical AF ablation is about 0-1.6% [8], [10], [17], [31]. Four patients died during follow-up period, but three of the patients did not die of adverse cardiovascular or cerebrovascular events. Risk of stroke is high in our study, poor compliance to anticoagulation therapy of few patients may account for this. The incidence of permanent pacemaker implantation in our patients was 2.88%, which in keeping with 0-5.77% risk of permanent pacemaker implantation after minimally invasive surgical ablation for AF [9], [10], [17], [18], [19]. Rate of procedure associated adverse events were similar between our study and previous data [8], [10], [17], [19]. However, the incidence of adverse events during follow-up is still higher in the present study, which probably mainly because 55 months follow-up in our study is obvious longer than 12 months follow-up in previous study [8], [10], [17], [19].

### Limitation

The study has several limitations. First, the limited numbers of patients in this single-center study may produce bias and other incomplete evidence, thus these results may not be generalized. Second, 24 h Holter monitoring is less effective for the detection of asymptomatic arrhythmias, compared with transtelephonic daily monitoring or implantable loop recorders. Furthermore, the transesophageal echocardiogram was not used during operation to confirm whether the LAA was completely removed before the end of surgery. Finally, since left side and right side GPs are interconnected, by always ablating right GP first there will always be fewer active GPs on the left. It may reduce the number of active GP ablating on the left side which may affect the long-term results.

## Conclusions

In selected patients with AF, minimally invasive surgical PVI combined with GP ablation for AF has a single-procedure success rate of 51.8% for paroxysmal AF, 28.2% for persistent AF, 28.6% for long-standing persistent AF at 60 months. Patients with AF duration≤24months, LA diameter<40 mm and no ERAF, had favorable long-term outcomes. Whether additional GP ablation is beneficial or not for long-term results is also not clear. Future work is needed to compare long-term results between PVI with GP ablation and without GP ablation. Meanwhile, surgical ablation is also associated with a certain amount of complications. These results may not be generalized as they were observed in a single institution and relative small sample size. The accurate long-term results after minimally invasive surgical ablation must be evaluated in further larger studies.
