# Correction: Long-Term Results of a Minimally Invasive Surgical Pulmonary Vein Isolation and Ganglionic Plexi Ablation for Atrial Fibrillation

**DOI:** 10.1371/annotation/c46d8841-f33e-45ea-9c17-b754e1017641

**Published:** 2014-01-03

**Authors:** Shuai Zheng, Yan Li, Jie Han, Haibo Zhang, Wen Zeng, Chunlei Xu, Yixin Jia, Jiangang Wang, Kequan Guo, Yuqing Jiao, Xu Meng

In the Funding Statement, the name of a funding organization is incorrect. The Funding Statement should read: "This study was partly supported by National Natural Science Foundation of China (Grant No. 81270215) and China Postdoctoral Science Foundation funded project (Grant No. 2013M530664). The funders had no role in study design, data collection and analysis, decision to publish, or preparation of the manuscript." 

